# Growth performance, jejunal morphology, disaccharidase activities, and sugar transporter gene expression in Langde geese as affected by the *in ovo* injection of maltose plus sucrose

**DOI:** 10.3389/fvets.2023.1061998

**Published:** 2023-01-26

**Authors:** Desheng Li, De Xin Dang, Han Xu, Haizhu Zhou, Yujie Lou, Xiao Liu, Yan Cui

**Affiliations:** ^1^College of Animal Science and Veterinary Medicine, Jinzhou Medical University, Jinzhou, China; ^2^Department of Animal Resources Science, Dankook University, Cheonan, Republic of Korea; ^3^College of Animal Science and Technology, Jilin Agricultural University, Changchun, Jilin, China; ^4^College of Animal Science and Technology, Institute of Animal Nutrition, Northeast Agricultural University, Harbin, China

**Keywords:** geese, *in ovo* injection, maltose, sucrose, disaccharide, jejunum

## Abstract

**Introduction:**

The vigorous metabolic activity of an embryo increases the risk of low energy supply during incubation. The lack of energy during this critical period will lead to the death of an embryo. To avoid this risk, the *in ovo* injection technique *in ovo* allows for the injection of energy substances into an embryo.

**Methods:**

This study investigated the effects of *in ovo* injection of maltose and sucrose (MS) *in ovo* on post-hatching growth performance, jejunal morphology and disaccharidase activities, and sugar transporter gene expression in Langde geese. A total of 300 fertilized eggs (115.75 ± 1.25 g) obtained from 3-year-old Langde geese were used in this study. The eggs were randomly assigned to two groups, and the difference between the two groups was whether 25g/L maltose and 25g/L sucrose (MS) dissolved in 7.5g/L NaCl were injected into the amnion on embryonic day 24. Each group had six replicates, which each replicate containing 25 eggs. The goslings were raised till day 28.

**Results and discussion:**

The results showed that the *in ovo* injection of MS increased final body weight, average daily gain (ADG), and feed efficiency. Additionally, MS injection improved post-hatching jejunal morphology, disaccharidase activities, and sugar transporter gene expression at an early stage. Therefore, we considered that the *in ovo* injection of MS had positive effects on the nutrient absorption capacity of goslings, thus contributing to the improvement in their growth performance.

## Introduction

Energy plays an important role in promoting organ development in birds (1). Under natural conditions, hatched birds can move freely in a short period of time and immediately receive exogenous energy substances (2, 3). However, modern poultry husbandry always adopts the strategy of “all-in and all-out,” that is, eggs fertilized with the same batch are hatched and removed at the same time (4). With the development of goose husbandry, the same strategy has been adopted. Although this strategy maximizes the benefits of poultry husbandry, it shows that early-hatched birds need to wait for later-hatched birds. Moreover, birds need to be vaccinated and packaged after hatching and undergo road transport to move to the farm. During this fasting period, nutrients to support the growth and development of birds depended on nutrients from the yolk sac (5, 6). However, the yolk sac contains only a small amount of energy substances. Additionally, the initial stage after incubation is a period in which the metabolic rate of birds is relatively high, increasing the energy demand (7). Therefore, birds face a shortage of energy supply in their early lives, and once the internal energy substances are depleted, they have to spend proteins to sustain life, which undoubtedly leads to the retardation of organ development (8–10). The intestine plays a central role in promoting growth, of which the jejunum is the core nutrient absorption site because it is rich in villi (11, 12). It has been reported that fasting affects the development of intestinal morphology (13).

The *in ovo* injection technique provides an opportunity to avoid the lack of energy in an embryo (14). An amnion is a suitable site for the delivery of nutrients to an embryo, which has been proven to be effective in promoting embryonic development (15). Amniotic fluid in an amnion surrounds the developing embryo, and birds start to imbibe amniotic fluid during the late-term embryonic development stage. *In ovo* injection can be used to provide nutrients or bioactive ingredients to optimize post-hatching growth performance (16), decrease the mortality rate (17), regulate energy metabolism (18), help the colonization of beneficial intestinal bacteria (19), improve intestinal health (20), and enhance nutrient absorption (21). Therefore, the *in ovo* injection of energy substances into an amnion seems to be an effective method to avoid the lack of energy supply in the early life.

It is reported that the *in ovo* injection of carbohydrates can improve the development of an embryo and therefore lead to higher growth performance and more mature intestinal morphologies in broiler chicks (22). However, studies focusing on the *in ovo* injection of carbohydrates for goslings are still limited. The future gosling breeding industry will benefit from learning more about *in ovo* nutrient injection. Maltose and sucrose (MS) are glucose precursors belonging to the disaccharide family, which can be used as exogenous energy donors to stimulate glucose anabolism, thus alleviating energy deficiency (23). Therefore, we hypothesized that the *in ovo* injection of maltose plus sucrose had positive effects on the growth performance, intestinal development, and nutrient absorption of goslings. The objective of this study was to investigate the effects of the *in ovo* injection of MS on growth performance, jejunal morphology, disaccharidase activities, and sugar transporter gene expression in Langde geese.

## Materials and methods

### Experimental design and sample collection

Fertilized eggs were obtained from Dekun Poultry Food Co., Ltd. (Meihekou, Jilin). To avoid inadequate number of eggs for the experiment caused by any nonexperimental factors-related embryo death, first, 500 fertilized eggs (122.97 ± 0.65 g) were obtained from 3-year-old Langde geese. Then, all freshly purchased eggs were incubated in a commercial incubator (Keyu CFZ microcomputer automatic incubator, Dezhou, Shandong). They were preheated and disinfected before incubation. The incubation program consisted of three stages:

Stage 1 (embryonic days 1–14): The temperature during this period was 38°C and the humidity was 65%.
Stage 2 (embryonic days 15–28): The temperature during this period was 37.5°C and the humidity was 55%.
Stage 3 (embryonic days 29–31): The temperature during this period was 37.2°C and the humidity was 70%.

All eggs were turned one time every 2 h for 180 s.

On embryonic day 23, we candled all eggs to ensure that the eggs used for injection contained live embryos. Then, a total of 300 fertilized eggs (124.32 ± 0.38 g) containing live embryos were selected and randomly assigned to two groups, with six replicates per group and 25 eggs per replicate. Eggs in the control group were not injected, but in the experimental group, they were injected with 25 g/L maltose and 25 g/L sucrose (MS) dissolved in 7.5 g/L NaCl. In the pre-experiment, we conducted a contrast experiment to compare the difference between the control group (non-injection) and the sham control group (injected with NaCl). No significant differences in growth performance, jejunal morphology, or jejunal disaccharidase activities were observed between the groups. Therefore, the sham control group was not designed in this experiment. The composition of the MS solution used in this study was determined based on our previous experiment (24).

The *in ovo* injection of MS was carried out on embryonic day 24. All injection solutions and paraffins were prepared before injection, which were sterilized at 121°C for 15 min and then allowed to reach room temperature (30°C). Before injection, eggs were cleaned in 70% ethanol and then put on a holder with a large end on top. The site of an amnion was identified by candling. An eggshell puncher was used to pierce the upper side of the eggs (the air space). A sterile syringe was used to inject the experimental solution (1.5 ml) into the amnion of the eggs with a depth of 20 mm. All eggs were held outside the incubator for < 5 min while being injected, including non-injected control eggs. Immediately after injection, the hole was sealed with paraffin and the eggs were returned to the incubator and incubated in accordance with the routine procedure until hatching.

After hatching, a total of 295 living birds were obtained, of which 147 were from the control group and 148 from the experimental group. Then, all living goslings were moved to a temperature-controlled room and assigned according to replicates. In this experiment, the sex of goslings was not determined. The goslings were raised in plastic-floored cages. Feeds were provided once they were moved to the cage (Table 1). All goslings were raised until day 28 with a uniform management program. The room temperature was maintained at 30°C for the first 3 days and then reduced by 2°C/week. This study was conducted under the supervision of the Animal Care and Use Committee of Jilin Agricultural University (Changchun, China).

**Table 1:** Composition and nutrient levels of the experimental basal diet (%, as-fed basis).

**Ingredients, %**	
Corn	60.00
Soybean meal	29.11
Wheat bran	6.00
Fish meal	2.00
Lysine-HCl	0.20
Methionine	0.23
Dicalcium phosphate	0.84
Limestone	0.82
Sodium chloride	0.30
Vitamin and trace mineral premix^a^	0.50
Total	100.00
**Calculated value, %**	
Metabolizable energy, MJ/kg	11.67
Available phosphorus	0.40
**Analyzed composition, %**	
Crude protein	19.78
Methionine	0.50
Total sulfate amino acid	0.77
Lysine	1.08
Calcium	0.78
Crude fiber	0.31
Neutral detergent fiber	1.09
Acid detergent fiber	0.35

^a^Provided per kilogram of complete diet: vitamin D_3_, 200 IU; vitamin A (retinyl acetate), 1,500 mg; vitamin E (DL-α-tocopheryl acetate), 12.5 mg; vitamin K_3_, 1.5 mg; thiamine, 2.2 mg; riboflavin, 5 mg; nicotinic acid, 65 mg; folic acid, 1 mg; pantothenic acid, 15 mg; pyridoxine, 2 mg; biotin, 0.2 mg; choline, 1,000 mg; Fe, 90 mg; Cu, 6 mg; Mn, 85 mg; Zn, 85 mg; I, 0.42 mg; Se, 0.3 mg; Co, 2.5 mg.

Goslings were sampled on the day of hatching, on day 7 post-hatching, and on day 28 post-hatching. Three birds were randomly selected at each sampling timepoint from each replicate cage, weighed, and slaughtered by cervical dislocation.

The jejunum sample was removed and cleaned with ice-cold saline. A segment of the jejunum approximately 1 cm long from the middle side was taken in duplicate and stored in two separate tubes. One of the samples was fixed with formalin solution (10% concentrations) for morphological analysis. Another sample was frozen in liquid nitrogen and stored at −80°C for measuring disaccharidase activities and sugar transporter gene expression.

### Analysis of experimental parameters

#### Growth performance

Cage-based body weight was measured on the day of hatching, day 7 post-hatching, and day 28 post-hatching to calculate average daily gain (ADG). Cage-based feed intake was measured daily for calculating average daily feed intake (ADFI). Feed efficiency was calculated as the ratio of ADG to ADFI.

#### Jejunal morphology

Jejunum segment samples were cut into small pieces to measure morphology according to the method described by Dang et al. (25). Small pieces of jejunum segment samples were fixed with 10% neutrally buffered formalin for 12 h and then dehydrated with alcohol of gradient concentration and xylene. The treated samples were then used to make paraffin blocks. A cryostat was used to make tissue sections. After removing paraffin, the samples were stained with hematoxylin and eosin. An optical microscope (Olympus, BX53F, Tokyo, Japan) was used to measure the values of villus height, width, and crypt depth at 10 × magnification. For each parameter, each slide was measured five times and represented as an average. The villus area was calculated by the villus height (from the villus tip to the junction of the villus crypt) and the width at half the height. Values given are the averages of 10 adjacent villi, and only vertically oriented villi were measured.

#### Jejunal disaccharidase activities

Jejunal segment samples were homogenized with 10 times the volume of cold normal saline. The homogenates were then centrifuged at 3,500 × g at 4°C for 15 min to collect the supernatant. According to the method described by Dang et al. (25), a colorimetric method was used to measure the activities of sucrase and maltase.

#### Jejunal sugar transporter gene expression

Based on the method described by Dang et al. (25), the RNAiso Reagent (TaKaRa, Dalian, Liaoning, China) was used to isolate the total number of ribonucleic acids (RNAs) from intestinal segment samples. The integrity and concentration of RNA were then determined. The primer sequences of the test gene were specially designed according to the sequences in GenBank (Table 2). Total RNA samples were purified using a specific kit and then reverse transcribed, followed by complementary deoxyribonucleic acid (cDNA) synthesis. Reverse transcription polymerase chain reaction was used to analyze the relative expression levels of sodium/glucose cotransporter protein-1 (SGLT-1), glucose transporter-2 (GLUT-2), and sucrase-isomaltase (SI) messenger RNA (mRNA) isolated from geese intestinal segment tissues. β-actin was used as an internal reference.

**Table 2:** Primers used for quantitative real-time polymerase chain reaction (PCR).

**Gene**	**Accession number^a^**	**Size (bp)^b^**	**Primer sequences (5**^**′**^ → 3^**′**^**)**	
			**Forward**	**Reverse**
SGLT-1	KU744842	126	GTAACATTGGCAGCGGACAT	TGGGTACAAACAGCCATCCT
GLUT-2	KU744841	118	CAGTTCTTCCTGCTCCTGCT	TCATCGGGTCACAGTTTCCT
SI	KU744844	193	CGTCACCTTCCCTCTTTGG	GGATTATGCTTCACTTCCACTTTG
β-Actin	M26111	158	GCCCAGCACGATGAAGAT	ATTTACGGTGGACGATGGAC

SGLT-1, sodium/glucose cotransporter protein-1; GLUT-2, glucose transporter-2; SI, sucrase-isomaltase.

^a^Accession number refers to Genbank (NCBI).

^b^PCR product size (base pairs).

#### Statistical analysis

Before the analysis, all the percentage data were transformed using arcsine transformations. The normality of the data was examined using the Shapiro–Wilk test andquantile–quantile (QQ) plots. The replicate cage (*n* = 6) served as the experimental unit. Student's *t*-test (SPSS 18.0 software) was used for multiple comparisons among treatments. The data are presented as means ± standard deviation (SD). The results were considered significant at a *p*-value of < 0.05.

## Results and discussion

In this study, the *in ovo* injection of MS led to increased body weight on the day of hatching (*p* < 0.001), on day 7 post-hatching (*p* < 0.001), and on day 28 post-hatching (*p* = 0.002) and ADG during days 1–7 post-hatching (*p* < 0.001), days 8–28 post-hatching (*p* = 0.035), and days 1–28 post-hatching (*p* = 0.006) (Table 3). The results obtained in this study were confirmed in a study by Foye et al. (26), who found that the *in ovo* injection of dextrin and maltose (20% dextrin and 3% maltose dissolved in 0.9% saline) increased post-hatching body weight in turkeys. Therefore, we considered that the *in ovo* injection of MS had positive effects on post-hatching growth performance in Langde geese. The improvement in feed efficiency is considered the main reason for improving growth performance (27). We also observed an improvement in the feed efficiency during days 1–7 post-hatching by the *in ovo* injection of MS (*p* = 0.007) (Table 3). Feed efficiency is defined as the ability of animals to convert feed nutrients into carcass composition. A high feed efficiency always corresponds to a high nutrient absorption capacity. Intestinal morphological development, the activities of intestinal digestive enzymes, and the expression levels of the intestinal nutrient transporter are important parameters reflecting nutrient absorption capacity. Wang et al. (28) found an improvement in post-hatching feed efficiency of broiler chicks induced by injecting fertile broiler eggs with exogenous nutrients, which was attributed to the improvement of small intestinal morphology. Gao et al. (29) reported that the *in ovo* injection of amino acids improved the feed efficiency of broiler chicks by increasing the activity of digestive enzymes in the intestine. Shehata et al. (30) conducted an *in ovo* injection experiment for broiler chicks and found that the *in ovo* injection of probiotics had positive effects on feed efficiency by upregulating the expression of nutrient transporter genes. Therefore, the improvement in feed efficiency seemed to be related to intestinal morphological development, the activities of intestinal digestive enzymes, and the expression level of an intestinal nutrient transporter.

**Table 3:** Post-hatching growth performance of goslings as affected by the *in ovo* injection of maltase and sucrose.

**Items**	**Control^a^**	**MS injection^b^**	***P*-value**
**Body weight, g**			
Day of hatching	96.40 ± 2.17	116.80 ± 2.12	< 0.001
Day 7 post-hatching	212.00 ± 35.19	283.50 ± 23.94	< 0.001
Day 28 post-hatching	1,294.80 ± 99.59	1,444.70 ± 109.80	0.002
**ADG, g**			
Days 1–7 post-hatching	16.52 ± 5.08	23.81 ± 3.41	< 0.001
Days 8–28 post-hatching	51.56 ± 3.67	55.30 ± 4.46	0.035
Days 1–28 post-hatching	42.80 ± 3.58	47.43 ± 3.92	0.006
**ADFI, g**			
Days 1–7 post-hatching	29.79 ± 9.89	33.43 ± 10.25	0.386
Days 8–28 post-hatching	126.40 ± 23.92	161.60 ± 59.09	0.076
Days 1–28 post-hatching	102.20 ± 19.85	129.60 ± 46.00	0.079
**Feed efficiency** ^c^			
Days 1–7 post-hatching	0.57 ± 0.09	0.77 ± 0.20	0.007
Days 8–28 post-hatching	0.42 ± 0.06	0.38 ± 0.13	0.416
Days 1–28 post-hatching	0.43 ± 0.06	0.41 ± 0.13	0.612

ADG, average daily gain; ADFI, average daily feed intake.

^a^Eggs without injected.

^b^Eggs injected with 25 g/L maltose and 25 g/L sucrose dissolved in 7.5 g/L NaCl.

^c^Gain to feed ratio.

An embryo injected with MS showed well-developed jejunal villi and a deep crypt depth. We observed that the *in ovo* injection of MS increased the villus height on the day of hatching (*p* = 0.044) and day 7 post-hatching (*p* = 0.022), the villus width on the day of hatching (*p* = 0.049) and day 7 post-hatching (*p* = 0.033), the villus surface area on the day of hatching (*p* = 0.042) and day 7 post-hatching (*p* = 0.032), the crypt depth on day 7 post-hatching (*p* = 0.011), and the ratio of the villus height to the crypt depth on day 7 post-hatching (*p* = 0.014). However, no statistical differences among the measured parameters were observed on day 28 post-hatching (Table 4). This indicated that the *in ovo* injection of MS can temporarily improve the morphology of the jejunum. The early development of intestinal morphology is important for optimizing the growth of poultry. Crypt depth and the height, width, and surface area of the villus are common parameters reflecting the morphological development of the intestine (31). Longer villus length, wider villus width, and a larger villus surface area make the nutrient absorption ability better. It is reported that the development of broiler chicks' jejunal villi can be improved by the *in ovo* injection of maltose (32). Chen et al. (33) reported that the *in ovo* injection of solutions composed of 25g/L maltose and 25g/L sucrose had positive effects on the length and surface area of jejunal villi in a duck. Therefore, we considered that the *in ovo* injection of MS had positive effects on the development of jejunal morphology, which partially contributed to the improvement in the early-stage post-hatching feed efficiency.

**Table 4:** Post-hatching jejunal morphology of goslings as affected by the *in ovo* injection of maltase and sucrose.

**Items**	**Control^a^**	**MS injection^b^**	***P*-value**
**Day of hatching**			
Villus height, μm	188.50 ± 13.01	221.40 ± 28.97	0.044
Villus width, μm	34.33 ± 5.57	40.91 ± 3.54	0.049
Villus surface area, μm^2^ × 10^3^	6.52 ± 1.50	9.13 ± 1.97	0.042
Crypt depth, μm	25.65 ± 4.53	28.21 ± 4.49	0.363
Villus height to crypt depth ratio	7.45 ± 0.82	7.87 ± 0.23	0.249
**Day 7 post-hatching**			
Villus height, μm	688.50 ± 41.00	795.30 ± 53.32	0.022
Villus width, μm	91.40 ± 4.55	113.60 ± 13.64	0.033
Villus surface area, μm^2^ × 10^3^	63.05 ± 6.88	90.83 ± 16.91	0.032
Crypt depth, μm	136.50 ± 8.14	187.10 ± 13.32	0.011
Villus height to crypt depth ratio	5.06 ± 0.22	4.27 ± 0.24	0.014
**Day 28 post-hatching**			
Villus height, μm	1,135.20 ± 143.40	1,307.40 ± 139.40	0.210
Villus width, μm	182.30 ± 18.17	192.40 ± 27.29	0.622
Villus surface area, μm^2^ × 10^3^	208.70 ± 27.02	254.10 ± 36.11	0.371
Crypt depth, μm	194.20 ± 26.28	230.70 ± 32.69	0.206
Villus height to crypt depth ratio	5.85 ± 0.05	5.69 ± 0.20	0.248

^a^Eggs without injected.

^b^Eggs injected with 25 g/L maltose and 25 g/L sucrose dissolved in 7.5 g/L NaCl.

Additionally, the increase in the activities of jejunal maltase and sucrase was observed on the day of hatching (*p* = 0.021) and day 7 post-hatching (*p* = 0.028), respectively. However, the activities of jejunal sucrase and maltase did not differ among groups on day 28 post-hatching (Table 5). Mature jejunal villi are always related to the secretion of more digestive enzymes. Digestive enzymes play an important role in the absorption of nutrients. Disaccharidases can decompose disaccharides into glucose, and the higher the activity of the intestinal disaccharide enzyme, the higher the content of glucose in it (34). Chen et al. (33) observed a higher jejunal sucrase activity when duck embryos were injected with a solution consisting of 25 g/L maltose and 25 g/L sucrose. Thus, we concluded that the *in ovo* injection of MS had positive effects on jejunal disaccharidase activities, which also contributed to the improvement in feed efficiency. However, similar to the improvement in jejunal morphology, the improvement in jejunal disaccharidase activities was also only observed in the early stages of post-hatching. Therefore, we considered that the *in ovo* injection of MS caused a temporary increase in disaccharidase activities in the jejunum *in ovo*.

**Table 5:** Post-hatching jejunal disaccharidase activities of goslings as affected by the *in ovo* injection of maltase and sucrose.

**Items**	**Control^a^**	**MS injection^b^**	***P*-value**
**Day of hatching**			
Sucrase, μmol·min^−1^·g^−1^ tissue	5.52 ± 1.41	6.25 ± 0.85	0.197
Maltase, μmol·min^−1^·g^−1^ tissue	1.08 ± 0.44	1.84 ± 0.71	0.021
**Day 7 post-hatching**			
Sucrase, μmol·min^−1^·g^−1^ tissue	8.11 ± 0.76	9.89 ± 1.30	0.028
Maltase, μmol·min^−1^·g^−1^ tissue	4.95 ± 0.79	6.08 ± 1.07	0.082
**Day 28 post-hatching**			
Sucrase, μmol·min^−1^·g^−1^ tissue	8.17 ± 1.01	9.66 ± 1.24	0.182
Maltase, μmol·min^−1^·g^−1^ tissue	4.69 ± 1.10	5.85 ± 1.07	0.258

^a^Eggs without injected.

^b^Eggs injected with 25 g/L maltose and 25 g/L sucrose dissolved in 7.5 g/L NaCl.

On the other hand, we observed that the *in ovo* injection of MS upregulated the expression of SGLT-1 on the day of hatching (*p* = 0.029) and day 7 post-hatching (*p* = 0.035), GLUT-2 on the day of hatching (*p* = 0.026) and on day 7 post-hatching (*p* = 0.027), and SI on the day of hatching (*p* = 0.024) *in ovo*. However, no statistical differences were observed among the measured parameters on day 28 post-hatching (Table 6). When disaccharide substances decompose into glucose, they are transported to enterocytes by specialized transporters. Among them, the expression of SI allows carbohydrates to be degraded into glucose, which could provide the supply of substrates for future nutrient absorption (35). Subsequently, glucose will be absorbed by SGLT-1, which is located apically in the intestinal epithelium, and then transported into the blood *via* basolateral membrane-expressed GLUT-2 (36, 37). Dong et al. (38) observed that the *in ovo* injection of MS (2.5% maltose and 2.5% sucrose dissolved in 0.75% saline) upregulated the expression of SGLT-1 and GLUT-2 in the jejunum in pigeons. Similarly, in this study, the embryos of Langde geese received MS injections, which upregulated the SGLT-1, GLUT-2, and SI genes in the jejunum. Similar to jejunal morphology and disaccharidase activities, the upregulation of jejunal sugar transportation genes was only observed on days 1 and 7 post-hatching and not on day 28 post-hatching. Therefore, we considered that the *in ovo* injection of MS had positive effects on the expression of sugar transporter genes; however, these effects only occurred at the early stage of post-hatching.

**Table 6:** Post-hatching jejunal sugar transporter gene expression of goslings as affected by the *in ovo* injection of maltase and sucrose.

**Items**	**Control^a^**	**MS injection^b^**	***P*-value**
**Day of hatching**			
SGLT-1	1.00 ± 0.47	1.28 ± 0.41	0.029
GLUT-2	1.00 ± 0.19	2.12 ± 1.75	0.026
SI	1.00 ± 0.28	1.63 ± 0.97	0.024
**Day 7 post-hatching**			
SGLT-1	2.59 ± 1.72	4.60 ± 0.57	0.035
GLUT-2	2.68 ± 1.22	4.97 ± 1.50	0.027
SI	1.99 ± 0.86	2.85 ± 0.33	0.062
**Day 28 post-hatching**			
SGLT-1	1.96 ± 0.89	3.12 ± 0.58	0.133
GLUT-2	1.72 ± 1.21	2.68 ± 0.29	0.253
SI	1.52 ± 0.42	2.07 ± 0.40	0.178

SGLT-1, sodium/glucose cotransporter protein-1; GLUT-2, glucose transporter-2; SI, sucrase-isomaltase.

^a^Eggs without injected.

^b^Eggs injected with 25 g/L maltose and 25 g/L sucrose dissolved in 7.5 g/L NaCl.

The results observed in this study showed that the improvement in feed efficiency was apparent only in the early stage post-hatching, which corresponded to improvements in jejunal morphology, jejunal disaccharidase activities, and jejunal sugar transporter gene expression. Thus, we considered that improved feed efficiency was related to improved nutrient absorption capacity by improving jejunal morphology, jejunal disaccharidase activities, and jejunal sugar transporter gene expression to improve growth performance.

## Conclusions

In conclusion, the *in ovo* injection of MS had positive effects on jejunal morphology, disaccharidase activities, and sugar transporter gene expression, which were considered as the reasons for improving feed efficiency and therefore improving growth performance. However, improvements in jejunal morphology, disaccharidase activities, and sugar transporter gene expression appeared only at the early stage of post-hatching, which indicated that the improvement in jejunal nutrient absorption capacity was temporary. In summary, the *in ovo* injection of MS had positive effects on the nutrient absorption capacity of goslings, therefore improving post-hatching growth performance.

## Data availability statement

The raw data supporting the conclusions of this article will be made available by the authors, without undue reservation.

## Ethics statement

The animal study was reviewed and approved by the Animal Care and Use Committee of Jilin Agricultural University (Changchun, China).

## Author contributions

DL and DD: writing—original draft, investigation, and writing—review and editing. HZ, YL, and XL: formal analysis and investigation. HX: conceptualization and methodology. DL and YC: supervision and writing—review and editing. All authors contributed to this article and approved the submitted version.
